# Band and Field Coengineered Charge Trap Memristor via Au Nanoparticle Layer for Programming Speed Enhancement

**DOI:** 10.1002/smsc.202500309

**Published:** 2025-10-16

**Authors:** Geunyoung Kim, Jiyul Park, Woojoon Park, Myeongchan Ko, Min Gu Lee, Hangyu Cho, Kyung Min Kim

**Affiliations:** ^1^ Applied Science Research Institute Korea Advanced Institute of Science and Technology (KAIST) Daejeon 34141 Republic of Korea; ^2^ Graduate School of Semiconductor Technology Korea Advanced Institute of Science and Technology (KAIST) Daejeon 34141 Republic of Korea; ^3^ Department of Materials Science and Engineering Korea Advanced Institute of Science and Technology (KAIST) Daejeon 34141 Republic of Korea

**Keywords:** artificial synapses, charge trapping, memristors, nanoparticles, self‐rectifying

## Abstract

Charge trap memristors (CTM) are promising candidates for nonvolatile analog units in crossbar array platforms, offering a pathway to next‐generation high‐density memory and synaptic arrays, due to their low‐current operation and self‐rectifying characteristics. However, the charge trapping process is inherently slow, posing a significant challenge to achieving high‐speed operation. In this study, a CTM device incorporating a gold nanoparticle (Au NP) layer, referred to as NP‐CTM, is proposed. This design improves programming speed by a factor of ≈47.6, while maintaining excellent stability and retention characteristics. These enhancements are attributed to the synergistic effects of enhanced electric fields and an engineered band structure, both achieved through the physical structure and material properties of Au NPs. The findings are validated through multiphysics‐based simulations and conduction band model analyses. The improved speed and the resulting reduction in programming energy of the CTM device make it a promising candidate for large‐scale applications.

## Graphical abstract

The Pt/Ta_2_O_5_/Nb_2_O_5−*x*_/Au nanoparticles/Al_2_O_3−*y*_/Ti charge trap memristor (CTM) demonstrates faster and energy‐efficient operation compared to its counterpart without Au nanoparticles. This improvement is attributed to the synergistic effects of localized electric field enhancement and optimized electron trapping via band engineering. The findings present a novel strategy for overcoming the inherent voltage–time dilemma of CTM devices.

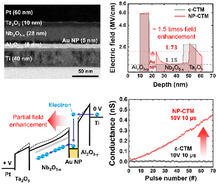

## 1 Introduction

Artificial intelligence technologies, which demand low latency and high energy efficiency for training, inference, and data generation based on massive datasets, are driving a fundamental shift in modern computing architectures from processor‐centric to memory‐centric models. In this context, memory is no longer a passive storage unit but an active element in the computational pipeline.^[^ ^1^, ^2^, ^3^ ^]^ One of the most promising directions in this paradigm is the integration of high‐density analog memory capable of high‐speed operation. These analog memories can represent a continuum of values, enabling more efficient implementation of neural operations, such as weighted summation and activation. Furthermore, the analog memory elements in a crossbar array platform can perform in‐memory computing tasks, thereby reducing the energy and time costs associated with data movement between memory and processing units.^[^ ^4^, ^5^ ^]^

A charge trap memristor (CTM) is a promising candidate for the analog nonvolatile memory, utilizing charge trapping and detrapping mechanisms similar to those employed in the operational principle of NAND flash memory. In contrast to the NAND device, the CTM device operates using only two terminals, enabling higher integration density, random access, and simplified operation. Furthermore, the CTM device does not require a forming process, resulting in exceptionally high uniformity. This forming‐free characteristic contrasts with other ion‐mediated memristive mechanisms, such as valence change memory (VCM) and electrochemical metallization (ECM), which require the forming process and therefore suffer from significant variability issues. Additionally, the CTM device exhibits a self‐rectifying behavior, eliminating the need for a selector to address leakage current issues in a large array integration.^[^ ^6^, ^7^, ^8^, ^9^ ^]^ Thanks to these advantageous characteristics, CTM devices hold potential as high‐density storage elements beyond NAND flash, where their inherent limitations can be minimized and their strengths can be fully leveraged.

However, the relatively high operating voltage and slow programming speed of the CTM device significantly undermine its competitiveness as a next‐generation memory technology. Previously, our group proposed the Pt/Ta_2_O_5_/Nb_2_O_5−*x*_/Al_2_O_3−*y*_/Ti structured CTM device, referred to as c‐CTM (denoting a conventional CTM device), operating at a programming operation voltage (*V*_PGM_) of ≈13 V.^[^ ^10^ ^]^ This *V*_PGM_ was lower than the typical *V*_PGM_ of NAND devices (≈20 V), but still higher than those of other mechanisms, such as the VCM and ECM, which operate near +1 V. Moreover, while the VCM and ECM memristors show sub‐microsecond programming speeds,^[^ ^11^, ^12^, ^13^, ^14^ ^]^ the CTM devices generally show programming speeds on the order of milliseconds. Although increasing the programming time can lower the required *V*_PGM_, this results in slower operation speeds and increased energy consumption. One straightforward approach to address this issue is to reduce the thickness of the tunneling layer (TL) and increase the tunneling probability. However, this approach may compromise the stability of the trapped charges, leading to adverse effects in terms of memory stability and retention (see Figure S1, Supporting Information, for the retention trend with different TL thicknesses). This represents the well‐known fundamental voltage–time dilemma in conventional memory technologies, and a fundamental solution is required to overcome this limitation.^[^ ^15^, ^16^ ^]^

**Figure 1:**
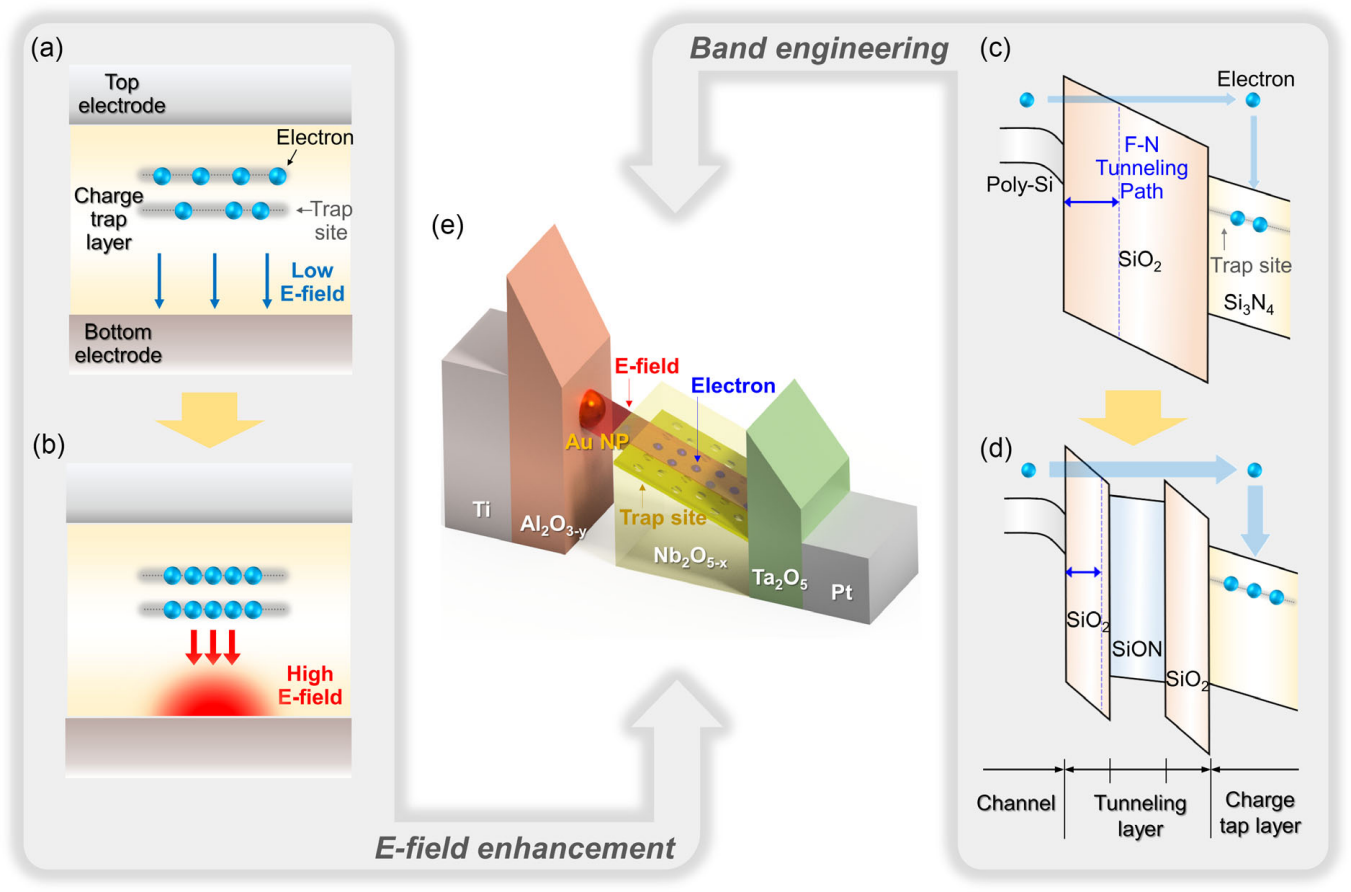
Concepts applied to the Pt/Ta_2_O_5_/Nb_2_O_5−*x*_/Au NPs/Al_2_O_3−*y*_/Ti (NP‐CTM) device. a) A nonfilamentary CTM structure exhibiting a lower uniform E‐field distribution. b) CTM with filament‐like confined structures exhibiting a higher local E‐field. c) Conventional TL structure of the NAND device. d) Band‐engineered TL structure designed to reduce the effective tunneling distance in a band‐engineered NAND device. e) NP‐CTM device incorporating Au nanoparticles that provides both enhanced E‐field and band engineering effects.

This challenge can be addressed by incorporating filament‐like confined structures^[^ ^17^, ^18^ ^]^ or through electronic band engineering.^[^ ^19^, ^20^, ^21^, ^22^ ^]^ As shown in **Figure** **1**a, the CTM's nonfilamentary structure offers the advantage of being electroforming‐free but inherently lacks the electric field (E‐field) enhancement provided by the filament structures. This is comparable to the VCM and ECM memristors, which leverage localized E‐field enhancement effects induced by filamentary structures, as depicted in Figure 1b. Applying a similar concept to the CTM device could potentially facilitate the programming process. Alternatively, as shown in Figure 1c,d, the NAND flash memory employs band engineering of the tunneling oxide to promote Fowler–Nordheim (F–N) tunneling for programming while maintaining direct tunneling for data retention. This approach could also be effective in the CTM device.

In this work, we propose a CTM device with an inserted NP layer (NP‐CTM), which incorporates both the E‐field enhancement effect and the band‐engineering effect, as illustrated in Figure 1e. The optimized NP‐CTM device adopts a Pt/Ta_2_O_5_/Nb_2_O_5−*x*_/Au NPs/Al_2_O_3−*y*_/Ti structure, in which the Au NP layer is inserted between the Nb_2_O_5−*x*_ charge trap layer (CTL) and Al_2_O_3−*y*_ TL of the c‐CTM device architecture. It exhibits a self‐rectifying ratio greater than 10^4^, an on/off ratio exceeding 10^3^, a maximum low resistance state (LRS) current below 10 μA, endurance over 10^5^, and retention exceeding 10^4^ s at 125 °C, all of which are comparable to the performance of the c‐CTM devices. Additionally, direct pulse measurements confirm that this device operates 47.6 times faster than previously reported c‐CTM devices at the same pulse conditions. We demonstrate that the combined effects of E‐field enhancement and band engineering are responsible for the observed performance gain, which are validated through COMSOL Multiphysics simulations and conduction mechanism analysis, respectively.

## 2 Results and Discussion

### 2.1 Electrical Characteristics of the NP‐CTM Device

**Figure 2:**
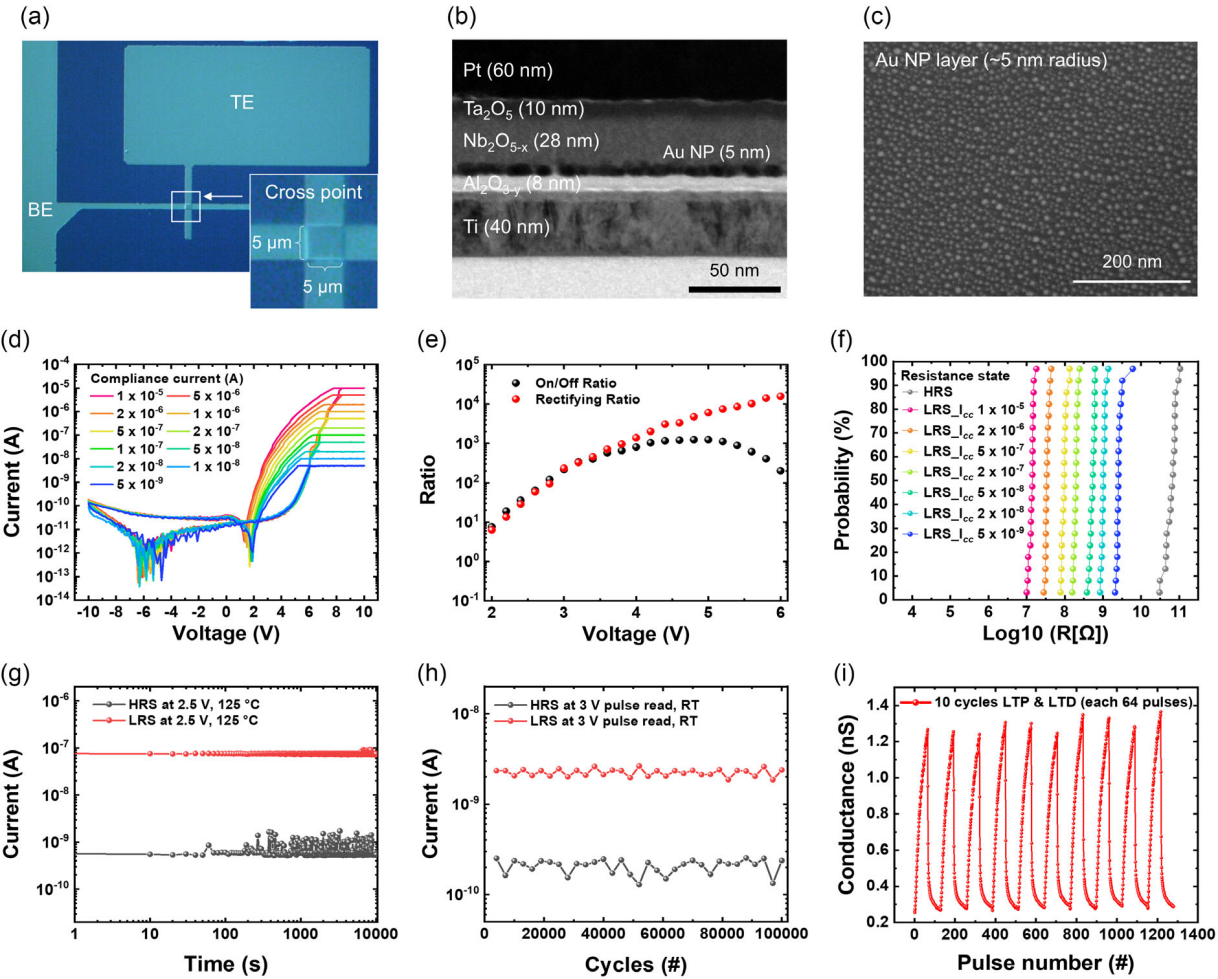
Cell characteristics of the NP‐CTM device. a) Top‐view optical microscope image of the device's cross‐point structure. b) Cross‐sectional TEM image of the device. c) SEM image of the Au NP layer on the Al_2_O_3−*y*_ after annealing at 200 °C for 30 min. d) Analog switching *I–V* curves of the device by controlling the compliance current (*I*_CC_) from 5 × 10^−9^ to 1 × 10^−5^ A. e) On/off ratio and rectifying ratio as functions of the applied read voltage. f) Distribution of resistance values at each *I*_CC_ level under a read voltage of +4.5 V across 20 cells. g) Retention characteristics up to 10^4^ s at 125 °C with a +2.5 V read operation. h) Endurance characteristics up to 10^5^ cycles, measured with +3 V read pulses at room temperature. i) LTP and LTD characteristics over ten operation cycles at a read voltage of +3 V.

**Figure** **2**a presents a top‐view optical microscope image of the cross‐point structure of the NP‐CTM device, with a cross‐point size of 5 ×5 μm. A cross‐sectional transmission electron microscopy (TEM) image in Figure 2b shows the device structure composed of 60 nm Pt/10 nm Ta_2_O_5_/28 nm Nb_2_O_5−*x*_/5 nm Au NPs/8 nm Al_2_O_3−*y*_/40 nm Ti. Figure 2c shows a scanning electron microscope (SEM) image of the Au NP layer on the Al_2_O_3−*y*_ after thermal treatment at 200 °C for 30 min to facilitate Ostwald ripening and coalescence, confirming the uniform distribution of the Au NP layer with a radius of ≈5 nm (see Figure S2, Supporting Information, for the atomic force microscope image of the Au NP layer).^[^ ^23^, ^24^, ^25^, ^26^ ^]^ Figure 2d shows analog *I–V* characteristics obtained at different compliance currents (*I*_CC_) ranging from 5 × 10^−9^ to 1 × 10^−5^ A, using a voltage sweep between −10 and +10 V. The pristine state of the device is the electroforming‐free high resistance state (HRS). The analog switching characteristic is attributed to the gradual charge trapping in the defect states of the CTL. As more charges become trapped, they generate a stronger internal negative field, which reduces the Schottky barrier height at the Ta_2_O_5_/Nb_2_O_5−*x*_ interface, leading to the LRS.^[^ ^10^ ^]^ Figure 2e plots the on/off ratio (=*I*_LRS_(@ + V)/*I*_HRS_(@ + V)) and the rectifying ratio (=*I*_LRS_(@ + V)/*I*_LRS_(@ − V)) as a function of the voltage at *I*_CC_ of 1 μA. The on/off ratio increases from +2 to +4.8 V, reaching a maximum value of 1.2 × 10^3^, and then gradually decreases. In contrast, the rectifying ratio shows a tendency to increase as the voltage increases, with a minimum value of ≈6.3 at +2 V and reaching 1.5 × 10^4^ at +6 V. This rectifying characteristic originates from the asymmetric interfacial barrier structure formed by the work function difference between the top and bottom electrodes, regardless of the presence of Au NPs, as in the case of c‐CTM.^[^ ^10^ ^]^ The demonstrated on/off ratio and rectifying ratio characteristics are sufficient to reliably operate a 4.4 Mbit crossbar array at a read voltage of +3 V without leakage current issues, demonstrating the potential of this device for large‐scale integration (see Figure S3, Supporting Information, for estimation of crossbar array size).^[^ ^27^, ^28^, ^29^ ^]^ Figure 2f illustrates the cumulative probability distribution of resistance values at each *I*_CC_ level under a read voltage of +4.5 V, which exhibits the maximum on/off ratio, across a total of 20 cells, suggesting sufficient uniformity to implement the triple‐level cell technology.^[^ ^30^, ^31^, ^32^ ^]^ Figure 2g illustrates the retention characteristics at 125 °C, read at +2.5 V (selected to ensure stability under high‐temperature conditions), showing a stable memory margin up to 10^4^ s. Figure 2h presents the endurance characteristics up to 10^5^ cycles, where the programming and erasing pulse conditions were +10 V for 100 μs and −10 V for 100 μs, and the reading pulse was +3 V for 1 ms. This on/off window corresponds to the mild switching condition of *I*_CC_ = 0.01 μA in Figure 2d, yet it sufficiently demonstrates the cycling stability of the device. Figure 2i shows the results of long‐term potentiation (LTP) and long‐term depression (LTD) characteristics during 64 pulses for each operation for artificial synapse applications, showing stable uniformity across 10 cycles at a read voltage of +3 V. For the LTP and LTD processes, the pulse amplitude and time were +10 V for 10 μs and −10 V for 100 μs, respectively, representing a significant improvement compared to the ≥1 ms required for the conventional c‐CTM device. A more detailed evaluation of the programming speed will be presented later.

### 2.2 Direct Monitoring of Programming Speed Based on the Position of the Au NP Layer

The degree of programming speed enhancement varied depending on the location of the NP layer. Here, we inserted the NP layer at three different positions with respect to the CTL, at the Nb_2_O_5−*x*_/Al_2_O_3−*y*_ interface (between the CTL and TL, i.e., identical to the NP‐CTM device), inside the Nb_2_O_5−*x*_ bulk (the center of the CTL), and the Ta_2_O_5_/Nb_2_O_5−*x*_ interface (between the blocking layer (BL) and CTL), and compared their switching characteristics.

**Figure 3:**
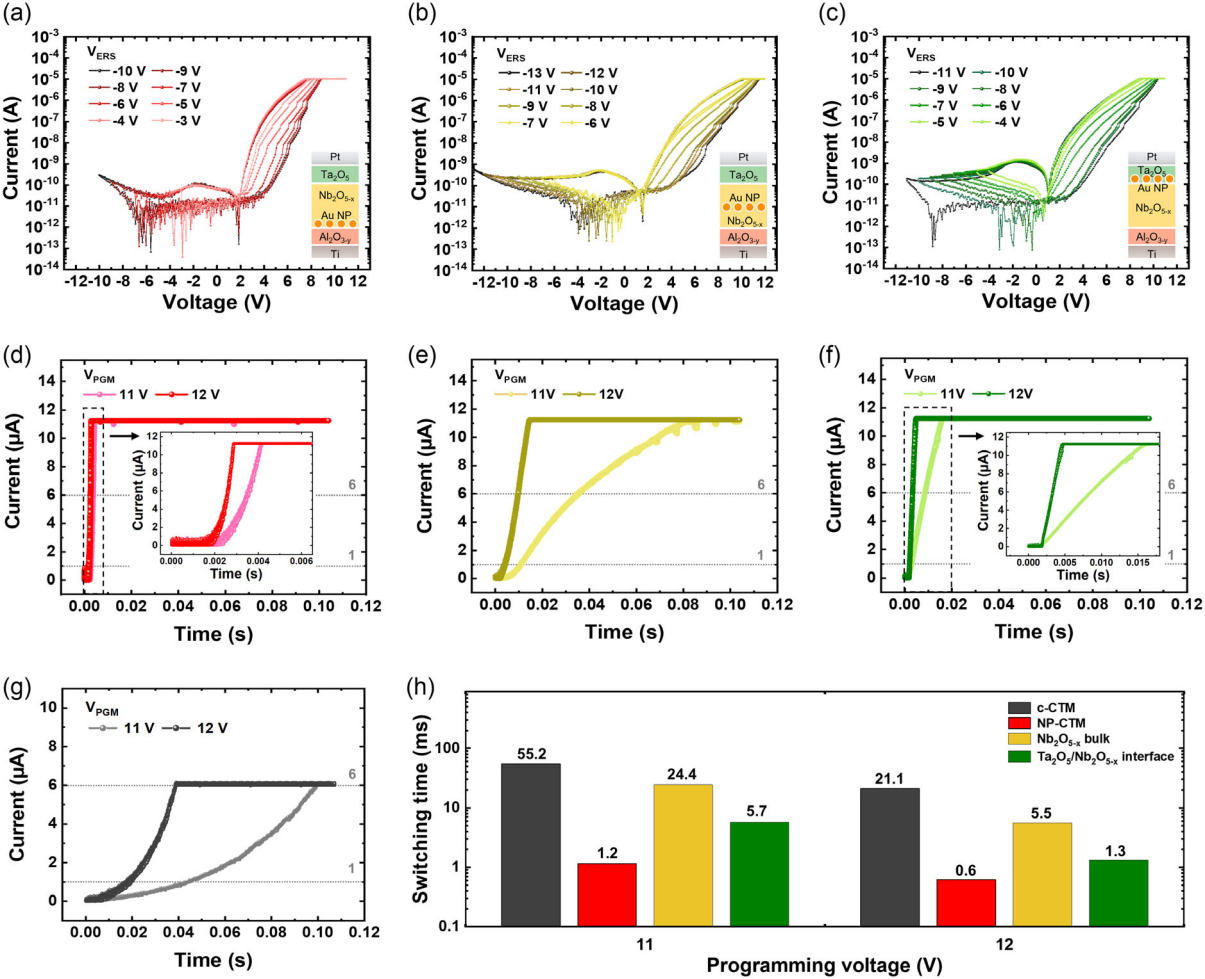
*I–V* curve characteristics and single programming pulse response based on the position of the Au NP layer. a–c) Analog erase characteristics of the device with the Au NP layer inserted at different positions: (a) Nb_2_O_5−*x*_/Al_2_O_3−*y*_ interface, (b) Nb_2_O_5−*x*_ bulk, and (c) Ta_2_O_5_/Nb_2_O_5−*x*_ interface. The insets show the device stack. d–f) Single programming pulse response for devices with the Au NP layer positioned at (d) Nb_2_O_5−*x*_/Al_2_O_3−y_ interface, (e) Nb_2_O_5−*x*_ bulk, and (f) Ta_2_O_5_/Nb_2_O_5−*x*_ interface, under pulse conditions of +11 and +12 V. g) Single programming pulse response for c‐CTM devices, under pulse conditions of +11 and +12 V. h) Comparison of the switching time required for the current to reach 6 from 1 μA during a single programming pulse in the c‐CTM device and NP‐CTM devices with different Au NP layer positions.

The results demonstrated an improvement in the programming speed in all three cases while maintaining the *I*–*V* characteristics. **Figure** **3**a–c shows the resistance switching *I–V* curves for the three cases, respectively. The insets show the device stack. Here, the *V*_PGM_ was fixed to +12 V with the *I*_CC_ of 10 μA while the erasing voltages (*V*_ERS_) were changed. Since the onset voltage of the reset operation differs for each device, a fixed step of −1 V was applied from the respective onset voltage to compare a total of eight analog reset characteristics. This was intended to further demonstrate the capability of analog erasing operations, in comparison to the analog programming operation shown in Figure 2d.

To measure the accurate switching speed of each device, we applied a sufficiently long single programming pulse and analyzed the response of the output current. This method directly reveals the dynamic change of the resistance state during the pulse duration. Note that this approach is applicable only to programming switching, which occurs in the forward direction. In the case of erasing switching, it takes place in the reverse direction and therefore cannot be directly observed using this method. Figure 3d–f presents the single *V*_PGM_ (+11 and +12 V) response for each device configuration. The pulse width was set sufficiently longer than the switching time for each measurement. Figure 3g shows the single *V*_PGM_ response of the c‐CTM device for reference. Through this measurement, we were able to monitor the dynamic resistance changes during the pulse duration and determine the switching time required for the device to reach a certain current level. Figure 3h summarizes the switching time required to reach 6 from 1 μA during a single *V*_PGM_ of +11 and +12 V, showing that the NP‐CTM device achieves faster switching speeds compared to the c‐CTM device (47.6 times faster at +11 V and 34.1 times faster at +12 V), based on the same current range.

**Figure 4:**
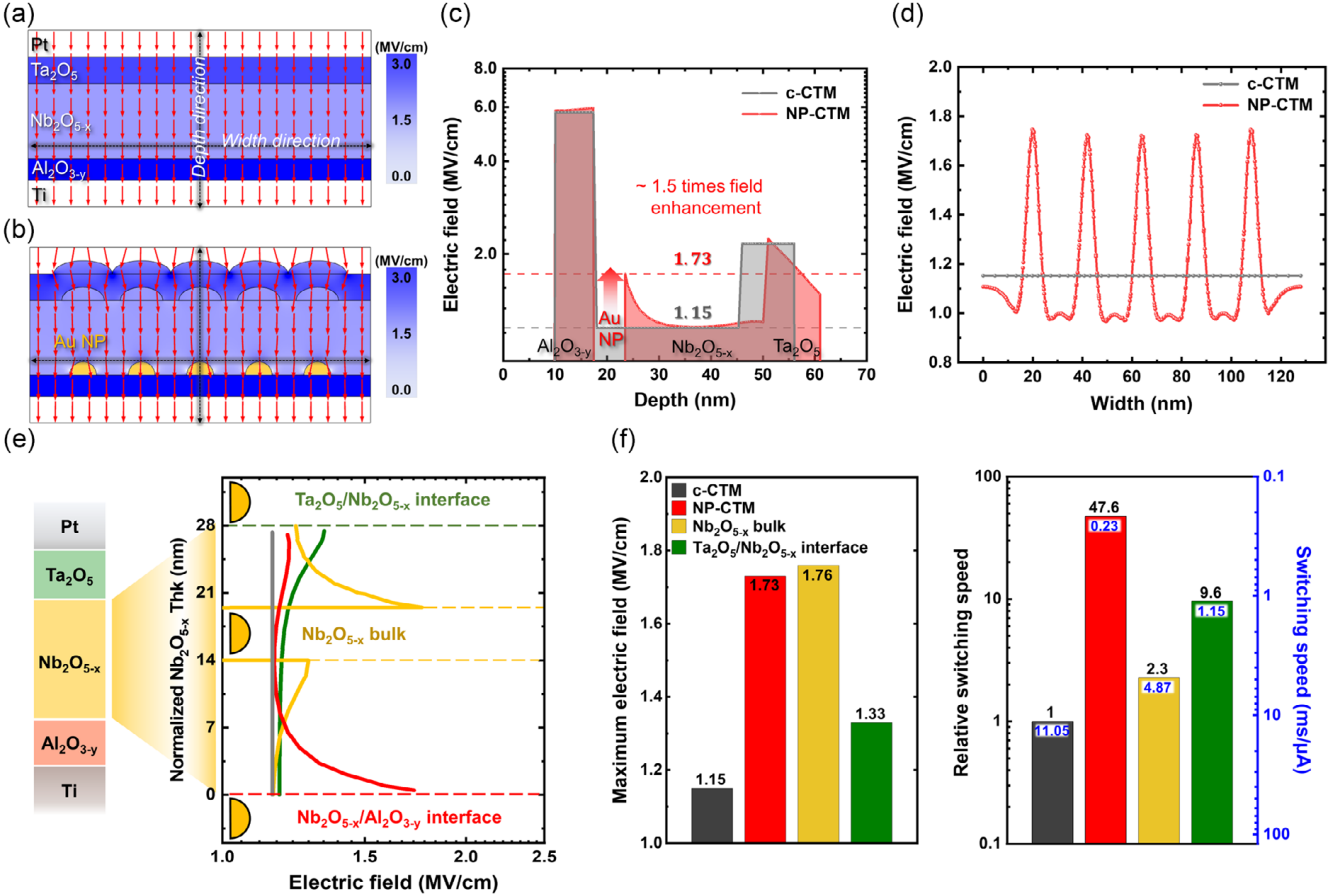
E‐field distribution of the c‐CTM device and NP‐CTM device simulated using COMSOL Multiphysics. a,b) Cross‐sectional simulation of the E‐field distribution in (a) the c‐CTM device and (b) the NP‐CTM device. c) Comparison of E‐field strength along the depth direction for the c‐CTM device (gray) and the NP‐CTM device (red). d) Comparison of E‐field strength along the width direction in the Nb_2_O_5−*x*_ for the c‐CTM device (gray line) and the NP‐CTM device (red line). e) E‐field profiles within the Nb_2_O_5−*x*_ under each condition. f) Comparison of the maximum E‐field in the Nb_2_O_5*−*x_ and the relative switching speeds, with the latter normalized to the c‐CTM. The right blue y‐axis indicates the switching speed, measured as the time (ms) needed for a 1 μA increase in current.

These results suggest that incorporating the NP layer may lead to an enhancement of the E‐field in all configurations.^[^ ^33^, ^34^, ^35^ ^]^ For further analysis, we conducted Multiphysics‐based simulations. Simulation details can be found in the Experimental Section. **Figure** **4**a,b represents the E‐field distribution in the c‐CTM device and NP‐CTM device, respectively, assuming a hemisphere shape of NP. The red arrows indicate the E‐field direction under a +11 V biased condition at the top electrode (see Figure S4, Supporting Information, for the E‐field distribution based on Au NP positions).

Figure 4c shows the E‐field distribution along the depth direction (marked as black arrows in Figure 4a,b), and Figure 4d compares the E‐field distribution along the width direction. When the Au NP layer was introduced, the E‐field became locally concentrated around the Au NPs, resulting in about 1.5 times enhancement in the E‐field intensity (the maximum E‐fields for the c‐CTM and NP‐CTM devices were 1.15 and 1.73 MV cm^−1^, respectively).

Figure 4e presents the E‐field profiles within the CTL during programming for the four device configurations, including both the NP‐CTM and c‐CTM. The E‐field enhancement effect was observed across all NP layer configurations. Figure 4f summarizes both the maximum E‐field values and the switching speeds (taken from Figure 3h). When comparing the maximum E‐field values and switching speed, however, no proportional relationship is observed. Moreover, the speed enhancement observed in the NP‐CTM is significantly greater than what can be expected based on E‐field enhancement alone. This indicates that the effect of Au NPs cannot be solely attributed to E‐field enhancement. Further explanation of this phenomenon will be discussed in the following sections.

### 2.3 Conduction Mechanism Analysis of the NP‐CTM Device

**Figure 5:**
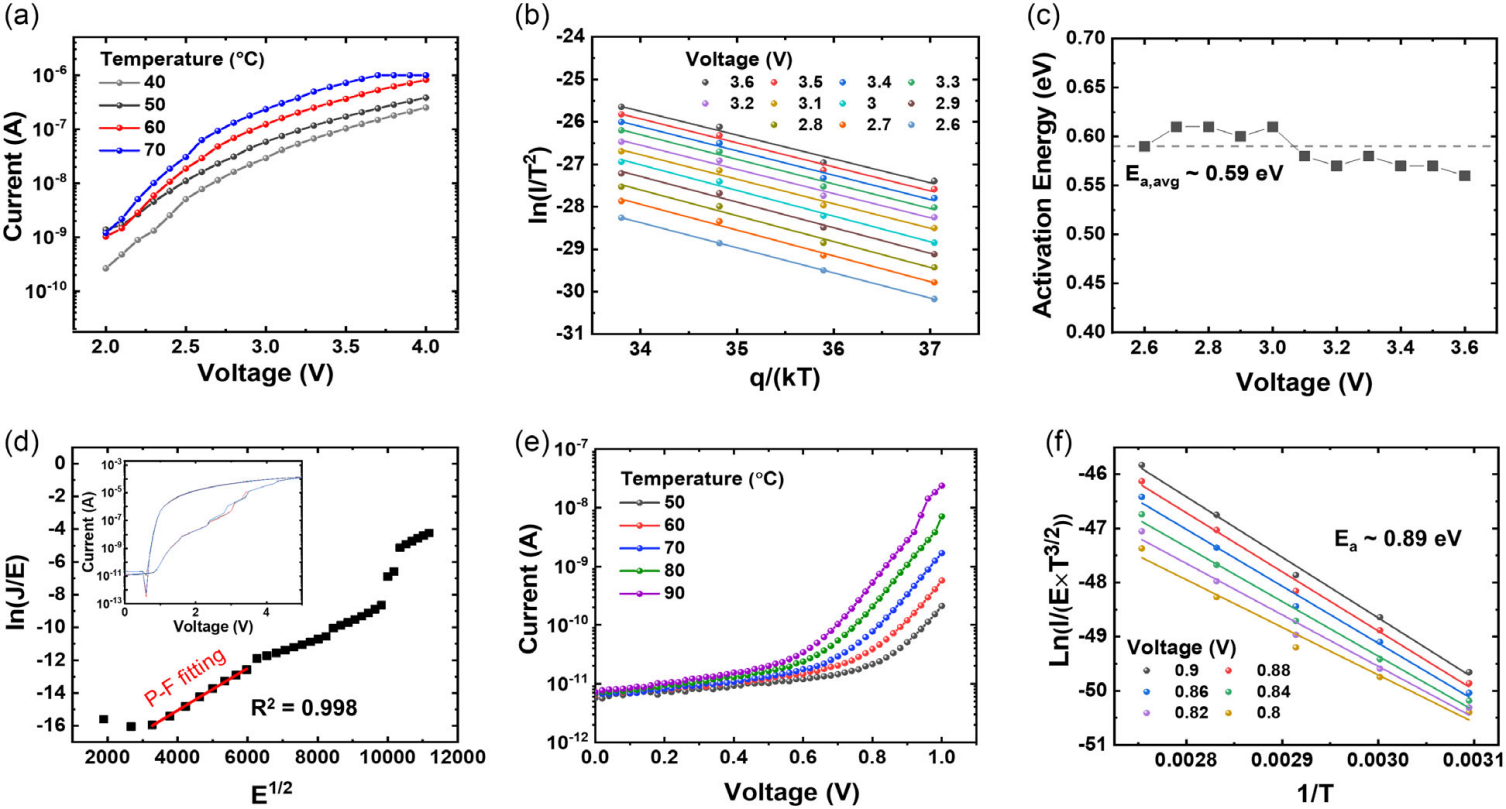
Conduction mechanism fitting and deep trap level analysis of the NP‐CTM device. a) LRS *I–V* curves at temperatures ranging from 40 to 70 °C. b) Schottky emission fitting (ln(*I/T* ^2^) versus *q*/*kT*) for voltage values between +2.6 and +3.6 V. c) Activation energy analysis for the NP‐CTM device. d) P–F emission form (ln(*J*/*E*) versus *E* ^1/2^) plot for the HRS after a full erase at −5 V in a single Nb_2_O_5−*x*_ layer device. The inset shows the *I–V* curve of the single Nb_2_O_5−*x*_ layer device after −5 V erase. e) HRS *I–V* curves at temperatures ranging from 50 to 90 °C. f) P–F emission fitting (ln(*I*/*E*×*T* ^3/2^) versus 1/*T*) for voltages between +0.8 and +0.9 V, used to calculate the deep trap energy level in the Nb_2_O_5−*x*_ CTL, determined to be 0.89 eV.

To fully understand the performance enhancement of the NP‐CTM device, it is essential to investigate its operational mechanism. In the previous study, the conduction mechanism of the c‐CTM device was identified as being dominated by Schottky emission at the barrier between the BL and CTL.^[^ ^10^ ^]^ It was observed that this barrier height decreases with charge trapping. To verify the NP incorporation effect, we performed conduction mechanism analysis of the NPCTM device and compared it to the previously proposed mechanism. **Figure** **5**a shows the LRS *I–V* curves measured at temperatures ranging from 40 to 70 °C. The NP‐CTM device exhibits a similar trend to the c‐CTM device, where the LRS current increases with increasing temperature.^[^ ^10^ ^]^ Based on these results, temperature‐dependent *I–V* curves in the voltage range of +2.6 to +3.6 V were plotted according to the Schottky emission equation (ln(*I/T* ^2^) versus *q*/(*kT*)), as shown in Figure 5b. This plot indicates that the Schottky emission remains the dominant conduction mechanism in the NP‐CTM device. The slope at each voltage is summarized in Figure 5c, which represents the activation energy (*E*_a_) required for electrons to transition from Nb_2_O_5−*x*_ to Ta_2_O_5_ in the LRS. The average *E*_a_ was 0.59 eV (0.56–0.61 eV), which is comparable to that of the c‐CTM device.^[^ ^10^ ^]^ These results indicate that the dominant conduction mechanism remains unaffected by incorporating the Au NP layer.

In the CTM devices, resistance switching occurs through charge trapping in deep trap states in the CTL, making the role of deep traps critical. Therefore, we next analyzed the deep trap levels of the CTL in the NP‐CTM device through the single CTL device fabrication (Pt/Nb_2_O_5−*x*_/Ti). Figure 5d shows the ln(*J/E*) versus *E* ^1/2^ plot for the Poole–Frenkel (P–F) emission fitting of the HRS. The P–F conduction of the HRS aligns well with the typically observed conduction in single amorphous Nb_2_O_5−*x*_ thin films.^[^ ^36^ ^]^ Figure 5e shows the HRS temperature dependence in the range of 50–90 °C. The deep trap energy level of the Nb_2_O_5−*x*_ was investigated using a ln(*I*/(*E×T* ^3/2^)) versus 1/*T* plot (temperature‐dependent P–F fitting) of the HRS, with a voltage range from +0.8 to +0.9 V, as shown in Figure 5f. The calculated *E*_a_ was 0.89 eV, slightly deviating from the reported 1.1 eV in the literature, but still considered to be within an acceptable value.^[^ ^37^ ^]^ Previous studies identified shallow trap levels located 0.22 eV below the conduction band (CB) of Nb_2_O_5−*x*_.^[^ ^38^ ^]^ This value is expected to be the same in the NP‐CTM device, as it utilizes the same Nb_2_O_5−*x*_ layer. Based on the experimentally identified trap energy level and band energy levels confirmed through reflected electron energy loss spectroscopy (REELS) and ultraviolet photoemission spectroscopy (UPS) analyses, we suggest a band diagram model of the NP‐CTM device as follows. In this model, interface‐related effects such as energy level offsets are not explicitly considered, as they are assumed to be inherently reflected in the experimentally extracted values (see Figure S5, Supporting Information, for REELS and UPS analysis results of the Nb_2_O_5−*x*_ layer).

**Figure 6:**
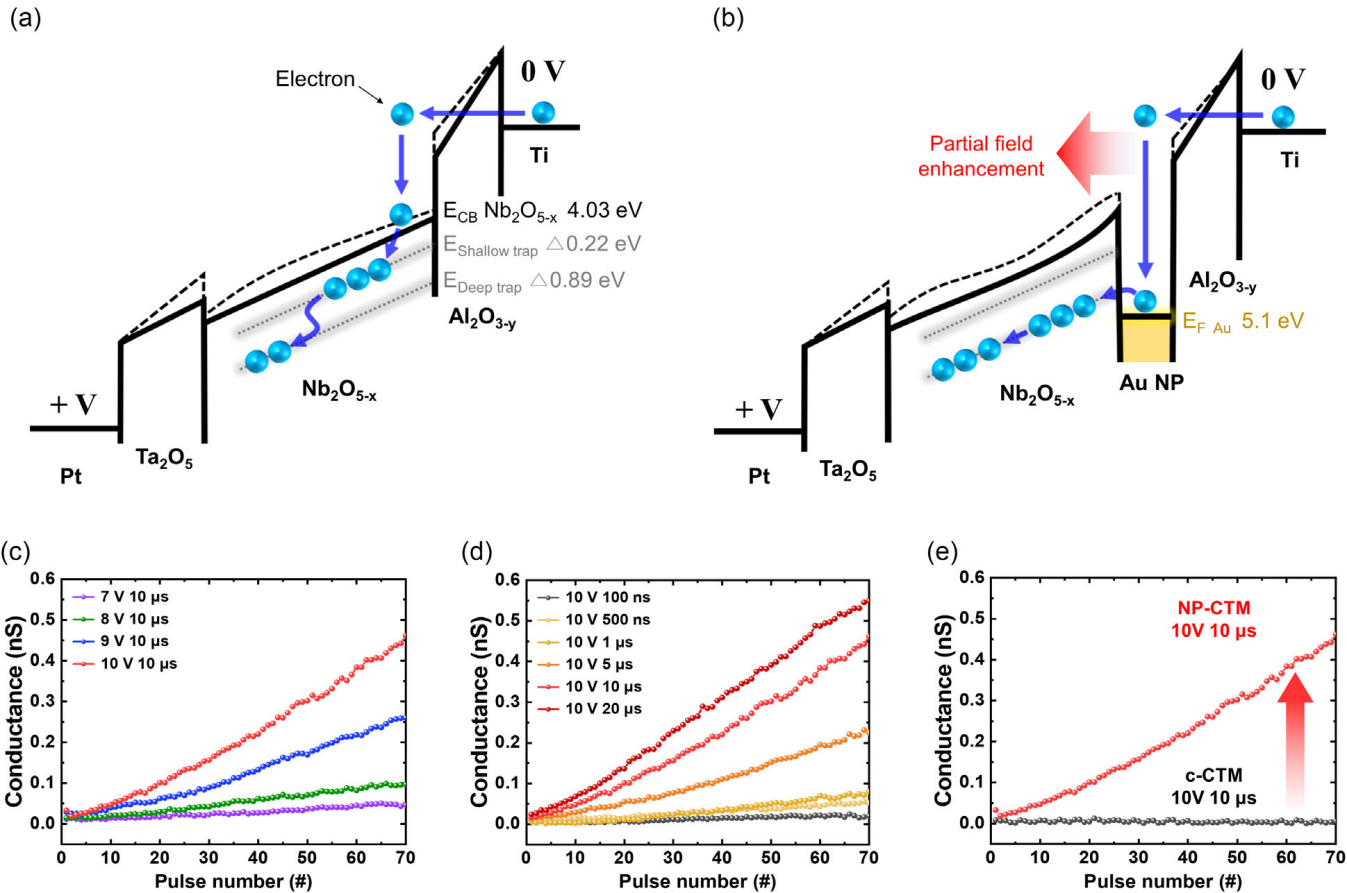
Comparison of the programming operation models and analog programming characteristics for the c‐CTM and NP‐CTM devices. a,b) Schematic energy band diagram of the programming operation model for (a) the c‐CTM device and (b) the NP‐CTM device. c,d) Conductance response of the NP‐CTM device (c) as a function of programming voltage, measured at a constant pulse width of 10 μs, and (d) as a function of pulse width, measured at a constant programming voltage of +10 V. e) Direct comparison of the multiprogramming pulse response between the c‐CTM device and NP‐CTM device.

**Figure** **6**a illustrates the band diagram of the c‐CTM device during the programming operation, proposed in our previous study.^[^ ^10^ ^]^ During this programming process, electrons pass through Al_2_O_3−*y*_ via F–N tunneling, reaching the CB (4.03 eV) of the Nb_2_O_5−*x*_. These electrons transit to shallow trap sites (4.25 = 4.03 + 0.22 eV) and eventually become trapped in the energetically most stable deep trap sites (4.92 = 4.03 + 0.89 eV) in the Nb_2_O_5−*x*_.^[^ ^39^ ^]^ This multistep transition process limits the programming time in the c‐CTM device. Figure 6b illustrates the band diagram of the NP‐CTM device during the programming operation. Similar to the c‐CTM device, electrons pass through Al_2_O_3−*y*_ via F–N tunneling. These electrons are first captured by the Au NPs (*E*_F_ = 5.1 eV). The *E*_F_ of Au is well aligned with the deep trap level of the Nb_2_O_5−*x*_, enabling direct injection into the deep traps while bypassing the shallow trap level, thereby reducing the programming time.^[^ ^40^, ^41^, ^42^ ^]^ If the Au NP layer is inserted within the CTL or at the BL/CTL interface, the charge transport process cannot bypass the shallow traps, rendering the band engineering effect ineffective (see Figure S6, Supporting Information, for a comparison of programming operation models based on Au NP positions in the CTM device).

Figure 6c shows the analog programming characteristics of the NP‐CTM device after applying multiple programming pulses by varying pulse amplitudes ranging from +7 to +10 V with a fixed pulse width of 10 μs. The conductance was read at +3 V. As the pulse amplitude increased, the conductance change rate also increased with excellent linearity. Figure 6d shows the conductance (read at +3 V) after applying multiple programming pulses by varying pulse widths ranging from 100 ns to 20 μs at a fixed pulse amplitude of +10 V. Similar to Figure 6c, as the programming pulse width increases, the conductance change rate becomes more pronounced. Figure 6e directly compares analog programming characteristics of the c‐CTM and NP‐CTM devices at the same programming pulse conditions, highlighting the charge trap boosting effect achieved by incorporating the Au NP layer (see Figure S7, Supporting Information, for more analog programming characteristics of the c‐CTM device with longer pulse width).

## 3 Conclusion

While CTM devices hold strong potential as storage elements beyond NAND flash, improving their relatively high operating voltage and slow switching speed is necessary for achieving more energy‐efficient operation. We demonstrated that the NP‐CTM device, incorporating an appropriately inserted Au NP layer, can significantly reduce both the programming voltage and time of the c‐CTM device, achieving ≈50‐fold enhancement in programming efficiency. This improvement results from the combined effects of enhanced E‐field localization and effective band engineering. Such an approach offers a novel solution to overcome the long‐standing voltage–time dilemma in conventional memory devices.

In terms of energy efficiency, the programming energy is significantly reduced from 503.36 pJ for the c‐CTM to 1.36 pJ for the NP‐CTM, highlighting a remarkable improvement (see Figure S8, Supporting Information, for the calculated programming energy based on the potentiation characteristics).

The introduced Au NP layer provided excellent benefits in terms of both structural configuration and energy band alignment, yet there remains substantial room for further optimization. In particular, alloying or other material modifications of the NP could enable more precise tuning of the Fermi energy levels to align more effectively with the deep trap levels. Through these continued efforts, we expect that CTM devices hold strong potential as next‐generation memory solutions.

## 4 Experimental Section

4.1

4.1.1

##### Fabrication of the NP‐CTM Device

For the device fabrication, a 40 nm‐thick Ti bottom electrode was evaporated and patterned by a lift‐off process on a SiO_2_(300 nm)/Si substrate. Then, the Al_2_O_3*−y*_ TL was deposited by thermal atomic layer deposition (ALD) at 250 °C using trimethylaluminum and H_2_O as the Al precursor and oxygen source, respectively. To form the Au NP layer, a thin Au layer was deposited with a slow deposition rate of 0.2 Å s^−1^ for 100 s on the Al_2_O_3*−*y_ TL using e‐beam evaporation. After deposition, an annealing process was conducted at 200 °C for 30 min to facilitate the nanoparticle formation. The Nb_2_O_5*−x*_ CTL was sputtered in a mixed Ar and O_2_ atmosphere using a Nb metal target at 200 °C. Next, the Ta_2_O_5_ blocking layer was deposited using plasma‐enhanced ALD at 225 °C. Tris(diethylamido)(tert‐butylimido)tantalum(V) (TBTDET) and O_2_ plasma were used as the Ta precursor and oxidant, respectively. Finally, a 60 nm‐thick Pt top electrode was deposited by e‐beam evaporation and patterned using a lift‐off process. The line width was 5 μm, so the device area was 25 μm^2^ with a crossbar structure.

##### Device Structure Analysis and Electrical Measurements

The cross‐section images were taken using an ultracorrected‐energy‐filtered TEM (Libra 200 HT Mc CS). For the electrical measurements, a semiconductor parameter analyzer (Keithley 4200A‐SCS) was used. During the electrical measurement, the top electrode was biased while the bottom electrode was grounded.

##### E‐Field Simulation

The E‐field simulation was performed using COMSOL Multiphysics. The simulation geometry was modeled based on the TEM images. The thickness of the Al_2_O_3*−y*_ layer was set to 8 nm with a dielectric constant of 9. The Nb_2_O_5*−x*_ CTL was modeled with a thickness of 28 nm and a dielectric constant of 45. The Ta_2_O_5_ blocking layer was set to 8 nm with a dielectric constant of 24. For the electrodes, both the bottom Ti layer and the top Pt layer were assigned a thickness of 10 nm. The embedded Au NPs were modeled as hemispherical structures with a radius of 5 nm and an interparticle distance of 12 nm.

##### Statistical Analysis

The cumulative probability data in Figure 2f were obtained from 20 cells. All statistical analyses were performed using Origin.

## Supporting Information

Supporting Information is available from the Wiley Online Library or from the author.

## Conflict of Interest

The authors declare no conflict of interest.

## Supporting information

Supplementary Material

## Data Availability

The data that support the findings of this study are available from the corresponding author upon reasonable request.
