# Supplementary material for: Band and Field Coengineered Charge Trap Memristor via Au Nanoparticle Layer for Programming Speed Enhancement
Source: Small Sci. 2025 Oct 16;5(12):e202500309. doi: 10.1002/smsc.202500309 (PMC12697761; doi:10.1002/smsc.202500309)
Supplement: Supplementary file 1 — Supplementary Material [file SMSC-5-e202500309-s001.pdf]

Supporting Information

Band and Field Co-engineered Charge Trap Memristor via Au Nanoparticle Layer for Programming Speed Enhancement

*Geunyoung Kim[†], Jiyul Park[†], Woojoon Park, Myeongchan Ko, Min Gu Lee, Hangyu Cho, and Kyung Min Kim**

G. Kim
Applied Science Research Institute
Korea Advanced Institute of Science and Technology (KAIST),
Daejeon, 34141, Republic of Korea

J. Park, H. Cho, K. M. Kim
Graduate School of Semiconductor Technology
Korea Advanced Institute of Science and Technology (KAIST),
Daejeon 34141, Republic of Korea.

W. Park, M. Ko, M. G. Lee, K. M. Kim
Department of Materials Science and Engineering
Korea Advanced Institute of Science and Technology (KAIST),
Daejeon 34141, Republic of Korea.

E-mail: km.kim@kaist.ac.kr

This PDF file includes:

Supporting Figures

Supporting Figures

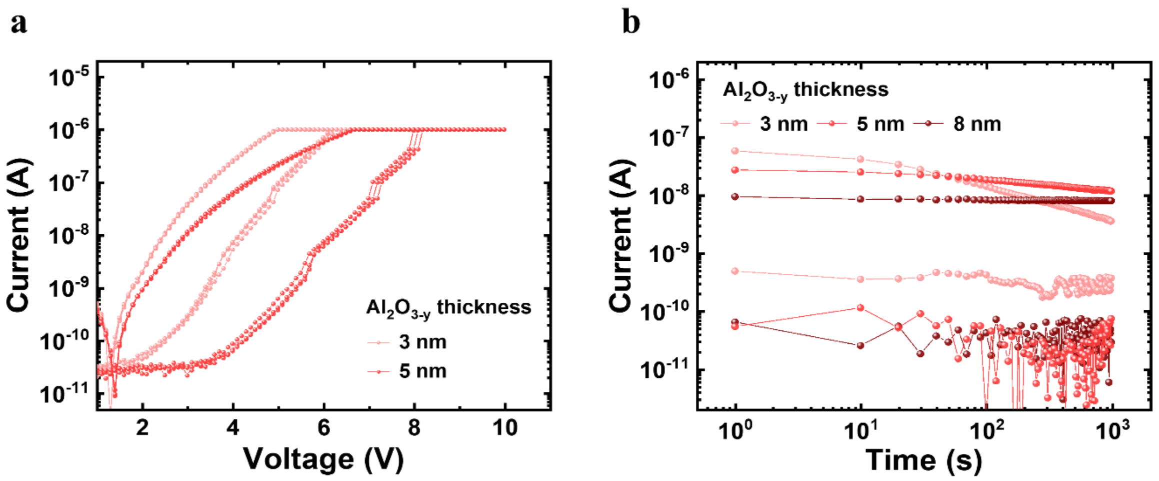

Figure S1. Cell characteristics of the Pt/Ta_2O_5/Nb_2O_{5-x}/Al_2O_{3-y}/Ti (c-CTM) device with different Al_2O_{3-y} thicknesses. a) The resistance switching *I-V* curves of the device by controlling the thickness of Al_2O_{3-y} at a compliance current (I_{CC}) of 1 μA. b) The retention characteristics up to 10^3 seconds by controlling the thickness of Al_2O_{3-y} at room temperature with 3 V read operation.

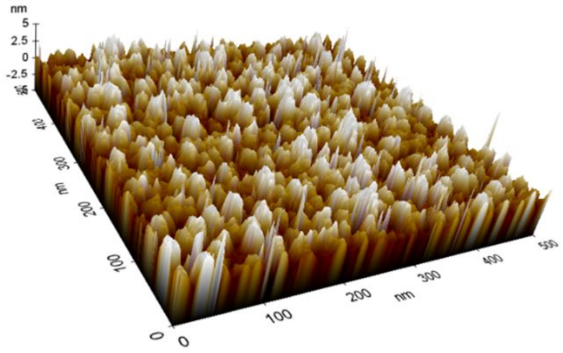

Figure S2. The atomic force microscope (AFM) image of the Au NP layer on the Al_2O_{3-y}. Root mean square roughness (Rq) is 1.2 nm. When nanoparticles are not present, it is 0.019 nm.

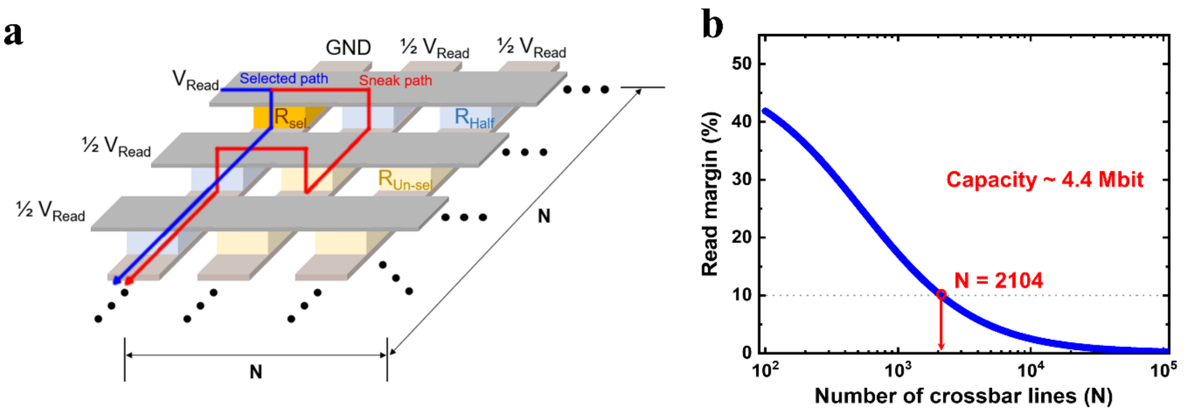

Figure S3. (a) Schematic illustration of sneak path currents in the N × N array. (b) Estimation of crossbar array size based on a 10% read margin criterion for read voltage +3 V. To calculate the maximum size and storage capacity of a crossbar array, the widely adopted one-bitline pull-up (OBPU) scheme was employed (S.-G. Ren et al., *IEEE Trans Electron Devices* 2021, 69, 838).[28]

$$R_{un} = \frac{2 \times R_{Half}}{N - 1} + \frac{R_{Un-sel.}}{(N - 1)^2} \tag{1}$$

$$Read\ Margin = \frac{\Delta V_{out}}{V_{pu}} = \frac{V_{out\_HRS} - V_{out\_LRS}}{V_{pu}} \tag{2}$$

$$V_{out\_HRS} = \frac{R_{sel\_HRS} \,//\, R_{un\_LRS}}{R_{sel\_HRS} \,//\, R_{un\_LRS} + R_{pu}} V_{pu} \tag{3}$$

$$V_{out\_LRS} = \frac{R_{sel\_LRS} \,//\, R_{un\_HRS}}{R_{sel\_LRS} \,//\, R_{un\_HRS} + R_{pu}} V_{pu} \tag{4}$$

$$R_{un\_LRS} = \frac{2 \times R_{Half\_LRS@^1/_2V_{Read}}}{N - 1} + \frac{R_{Un-sel.\_LRS@-V_{Read}}}{(N - 1)^2} \tag{5}$$

$$R_{un\_HRS} = \frac{2 \times R_{Half\_HRS@^1/_2V_{Read}}}{N - 1} + \frac{R_{Un-sel.\_HRS@-V_{Read}}}{(N - 1)^2} \tag{6}$$

All possible current paths, including the selected and sneak paths, can be equivalently represented as a combined parallel-series resistive circuit. Based on this, the total sneak path resistance, R_{un}, is defined as in equation (1). The read margin, which quantifies the signal distinction between the HRS and LRS, is defined by equation (2) as the ratio of the voltage difference across the selected cell in HRS and LRS states to the pull-up voltage (V_{pu}, equal to V_{Read}). The output voltage (V_{out}) is determined by the parallel connection of the cell resistance and sneak path resistance, and this parallel network is in series with the pull-up resistor (R_{pu}) applied to the selected word line. The output voltages in HRS and LRS are expressed by equations (3) and (4), respectively. The corresponding sneak path resistances in HRS and LRS conditions are calculated differently depending on the state, as defined by equations (5) and (6). Through these equations, the read margin can be analytically estimated by taking into account both the self-rectifying behavior and nonlinear characteristics of the memory device.

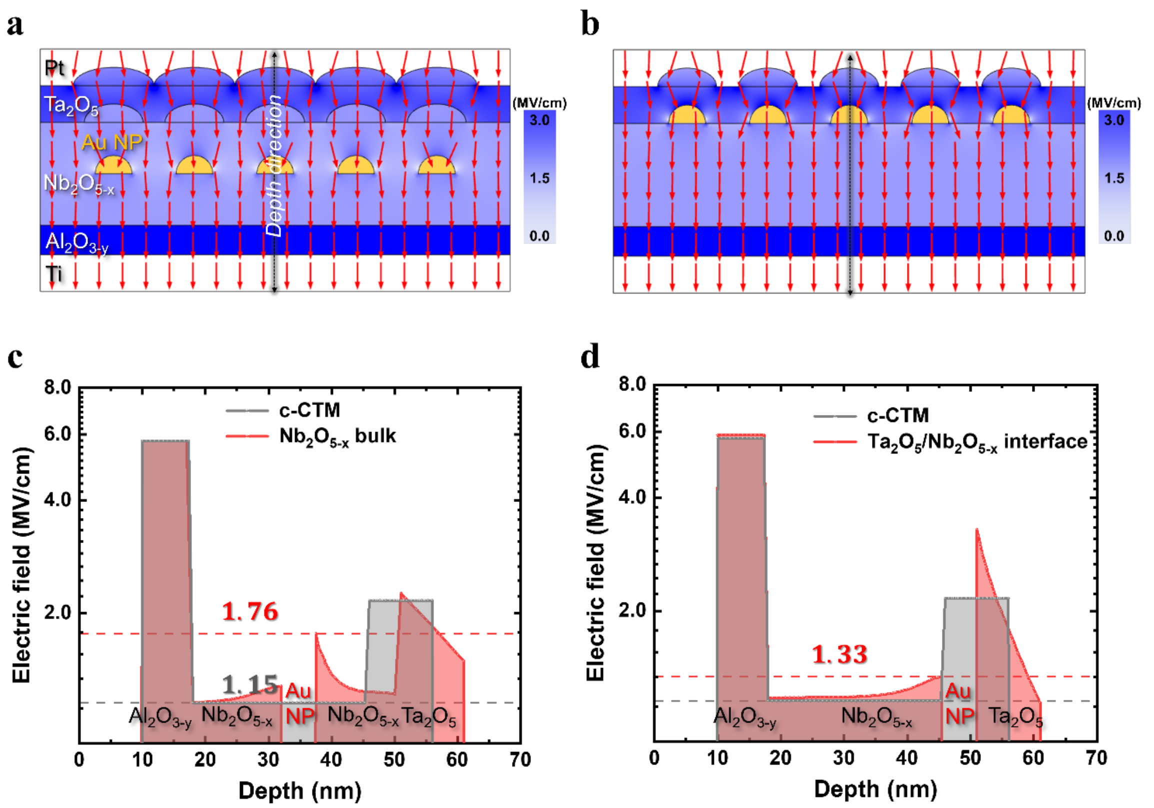

Figure S4. Electric field (E-field) distribution based on the Au NP position in the c-CTM device, simulated using COMSOL Multiphysics. Cross-sectional simulations of the E-field distribution in the device with the Au NP layer inserted at different positions: a) Nb_2O_{5-x} bulk and b) at Nb_2O_{5-x}/Ta_2O_5 interface. Comparison of E-field strength along the depth direction for the c-CTM device (gray) and the CTM devices (red) with the Au NP layer positioned at c) Nb_2O_{5-x} bulk and d) Ta_2O_5/Nb_2O_{5-x} interface.

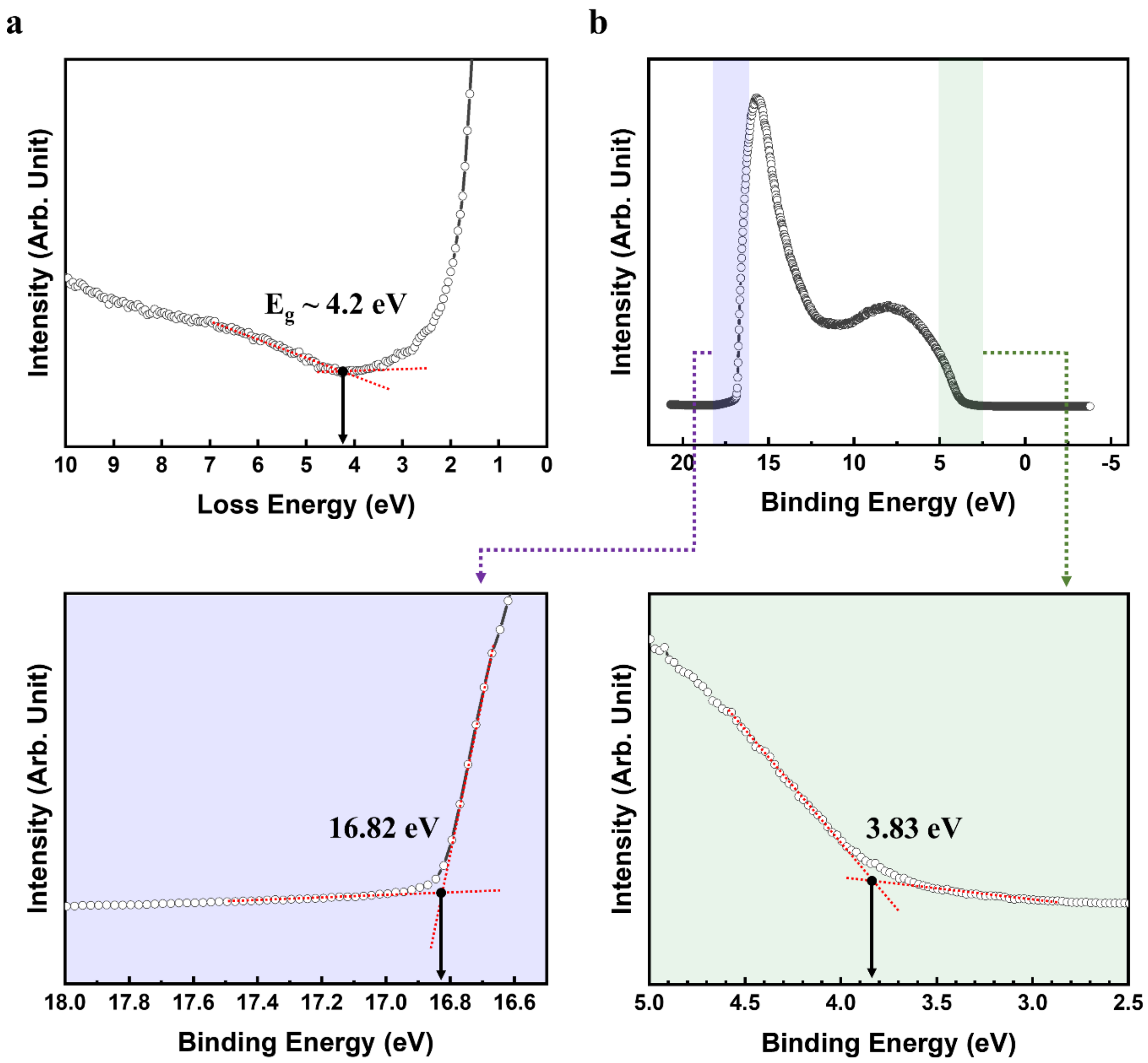

Figure S5. REELS & UPS analysis results of the Nb_2O_{5-x} layer. a) REELS spectrum for determining the band gap (E_g). b) UPS spectra used to calculate the work function (φ) and electron affinity (χ). The equation used for the χ value extraction is as follows:

$$\varphi = h\omega - E_{high\ cutoff} \quad (1)$$

$$\chi = \varphi - (E_g - E_{low\ cutoff}) \quad (2)$$

, where $h\omega$ is the photo energy of light (He UV emission at 21.22 eV), and E_{cutoff} values were obtained by fitting the energy graph of the UPS at high and low binding energies. The calculated E_g and χ values of the Nb_2O_{5-x} layer are 4.2 eV and 4.02eV, respectively.

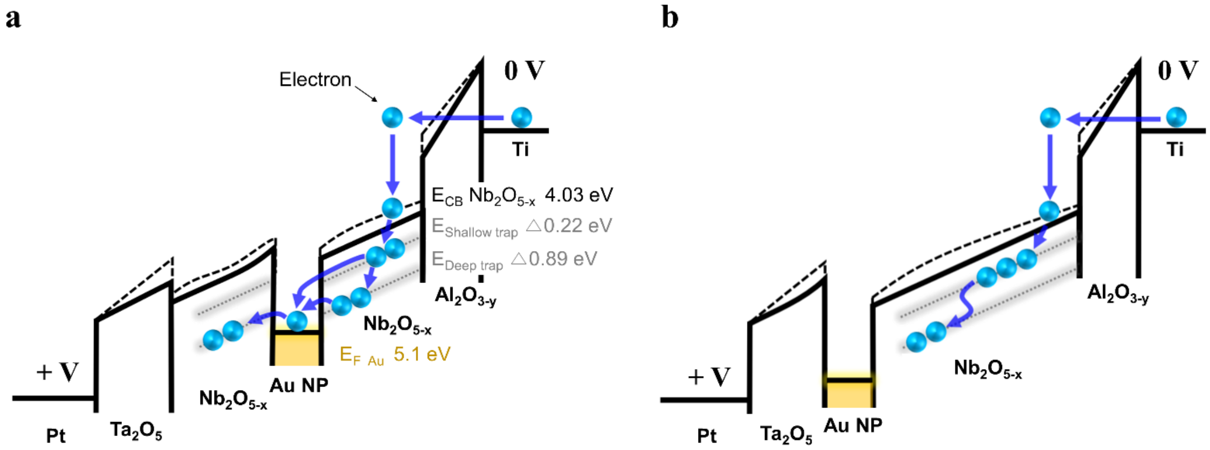

Figure S6. Comparison of the programming operation models based on the Au NP position in the CTM device. (a) Schematic energy band diagram of the programming operation model for the device with the Au NP layer inserted in the Nb_2O_{5-x} bulk. When the Au NP layer is positioned in the Nb_2O_{5-x} bulk, the electrons tunnel through the Al_2O_{3-y} layer and sequentially transition from shallow traps to deep traps, following the same charge transport pathway as in the c-CTM device. Thus, this configuration cannot offer band engineering advantage. The observed speed enhancement, approximately 2.3×, closely matches the E-field enhancement factor of 1.76, indicating that the improvement is primarily due to the increased electric field. (b) Schematic energy band diagram of the programming operation model for the device with the Au NP layer inserted at the Ta_2O_5/Nb_2O_{5-x} interface. When the Au NP is positioned at the Nb_2O_{5-x}/Ta_2O_5 interface, no additional band engineering effect can be expected, similar to the previous case. However, in this case, the speed enhancement (a factor of 9.8) is greater than the E-field enhancement (a factor of 1.33), indicating that additional factors may be contributing.
To explain this result, we propose the following plausible scenario: it is likely that Au NPs located at the Ta_2O_5/Nb_2O_{5-x} interface act as charge storage nodes, functioning similarly to floating gates. The upper region of the Nb_2O_{5-x} layer becomes more oxidized to Nb_2O_5 due to the oxygen supply from the ambient environment or the subsequent Ta_2O_5 ALD process, thereby reducing the density of oxygen vacancies. Additionally, when the Ta_2O_5 layer fully encapsulates the Au NPs, they can become electrically isolated, enhancing their role as charge storage floating gates. The presence of such charge storage sites may facilitate easier programming, which could account for the significant improvement in programming speed.

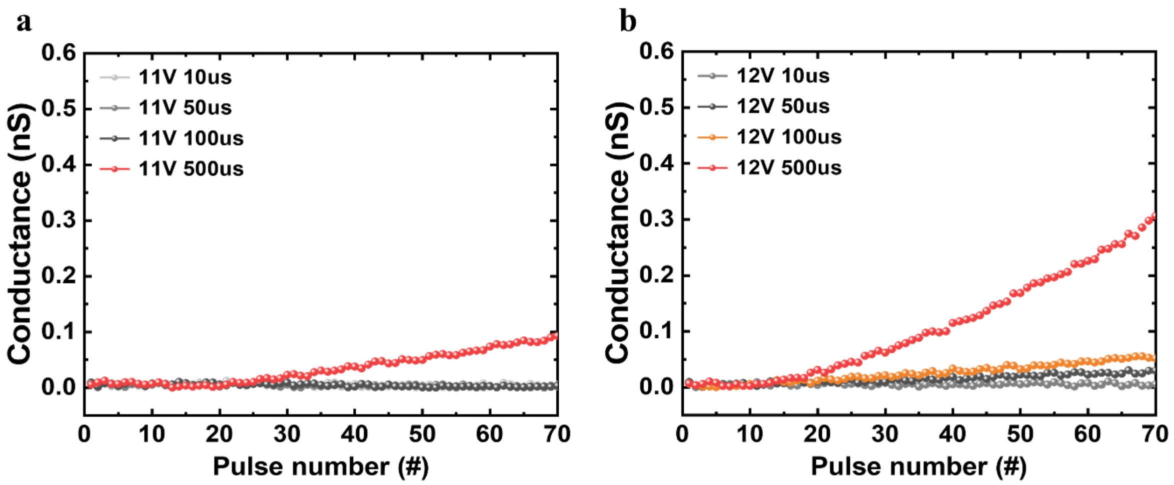

Figure S7. a,b) Conductance response of the c-CTM device as a function of pulse width, measured at a constant programming voltage of (a) +11 V and (b) +12 V. Without the Au NP layer, the device exhibited a conductance response at pulse widths longer than 500 µs.

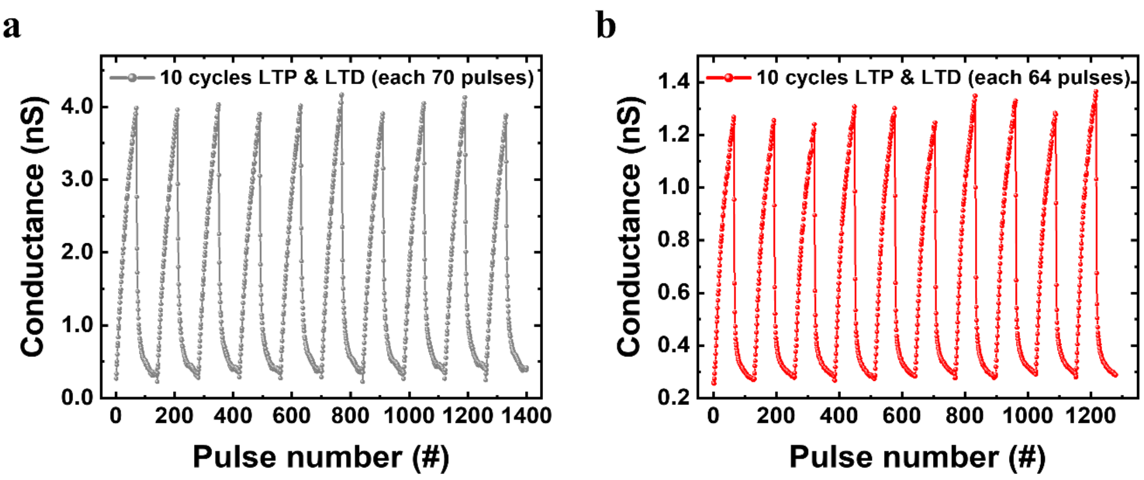

Figure S8. a,b) Long-term potentiation (LTP) and long-term depression (LTD) characteristics over 10 operation cycles for the c-CTM device (a) and the NP-CTM device (b). The c-CTM device exhibits LTP under a +11 V, 1 ms condition and LTD under a −9.5 V, 1 ms condition, whereas the NP-CTM device shows LTP at +10 V, 10 µs and LTD −10 V, 100 µs. The equation used for calculating energy per bit in maximum and minimum conductance states is as follows:

$$E = G \times V^2 \times t$$

, where G is the maximum and minimum conductance, V is the programming and erasing pulse voltage, and t is the programming and erasing pulse duration. Based on this definition, the calculated programming and erasing energy per bit in both the maximum and minimum conductance states is summarized as follows.

Operation	***Device***	***G***		***V***	***t***	***E***
Programming	c-CTM	Max.	4.16 nS	11 V	1 ms	503.36 pJ
	NP-CTM	Max.	1.36 nS	10 V	10 µs	1.36 pJ
Erasing	c-CTM	Min.	0.22 nS	−9.5 V	1 ms	20.33 pJ
	NP-CTM	Min.	0.26 nS	−10 V	100 µs	2.67 pJ
